# Pd Nanocatalyst Adorning Coral Reef Nanocomposite for the Synthesis of Nitriles: Utility of *Cucurbita pepo* Leaf Extract as a Stabilizing and Reducing Agent

**DOI:** 10.3390/nano9040565

**Published:** 2019-04-07

**Authors:** Mahmoud Nasrollahzadeh, Fatemeh Ghorbannezhad, S. Mohammad Sajadi, Rajender S. Varma

**Affiliations:** 1 Department of Chemistry, Faculty of Science, University of Qom, Qom 37185-359, Iran; ghorbannejhad69@gmail.com; 2 Department of Petroleum Geoscience, Faculty of Science, Soran University, P.O. Box 624 Soran, Kurdistan Regional Government, Iraq; phytochem2006@gmail.com; 3 Regional Centre of Advanced Technologies and Materials, Department of Physical Chemistry, Faculty of Science, Palacky University, Šlechtitelů 27, 783 71 Olomouc, Czech Republic

**Keywords:** cyanation, aryl halide, Pd/coral reef nanocomposite, *Cucurbita pepo*, Pd nanoparticles

## Abstract

A simple procedure for the palladium-catalyzed cyanation of aryl halides is described via a nucleophilic non-toxic cyanide source, K_4_[Fe(CN)_6_] in the presence of Pd/coral reef nanocomposite as a heterogeneous catalyst; the protocol provides a useful and easy method for the synthesis of aryl nitriles that are generated from the corresponding variant aryl halides, with sodium carbonate as a base. The nanocatalyst was prepared by a biological process using aqueous extract of leaves of *Cucurbita pepo* as a stabilizing and reducing agent and coral reef as a natural support, without deploying any hazardous chemicals. The catalyst, that is easily separable from the reaction mixture and reused multiple times, was characterized by FT-IR (Fourier-Transform Infrared Spectroscopy), ICP-AES (Inductively Coupled Plasma Atomic Emission Spectroscopy), XRD (X-ray Diffraction), TEM (Transmission Electron Microscopy), FE-SEM (Field Emission Scanning Electron Microscopy), EDS (Energy Dispersive X-ray Spectroscopy) and elemental mapping.

## 1. Introduction

**Scheme 1:**
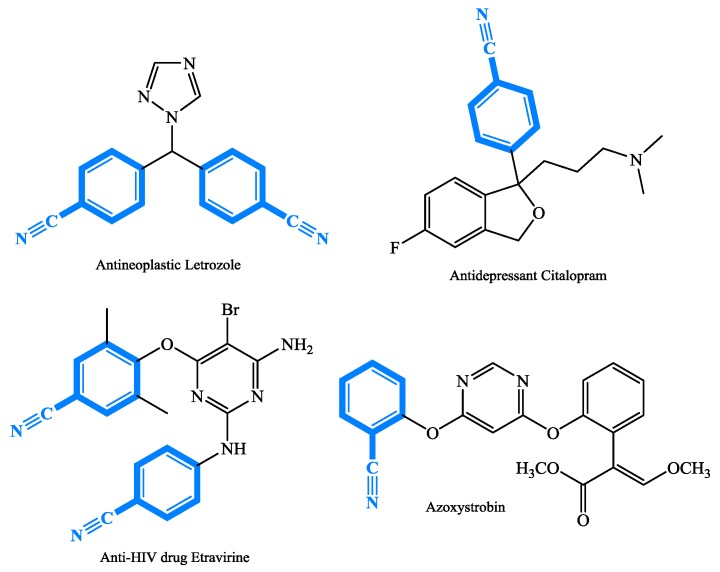
Selected examples of pharmaceuticals containing benzonitriles as the integral part.

Nitriles are implicated in the syntheses of a variety of organic compounds, and thus occupy a key position in organic chemistry manipulations. In the chemical industry, nitriles are oftentimes found as an impartible part of dyes, natural products, pharmaceuticals, agricultural and materials [1,2,3]. Aromatic nitriles have been utilized in diverse fields as both synthetic intermediates, as well as a final product in their own right, as shown by few examples in Scheme 1 [4]. Historically, two classical methods have been used for the introduction of a cyano group on to the aromatic ring: diazotization of anilines followed by a Sandmeyer reaction [5,6] with superstoichiometric amounts of copper(I) cyanide and Rosenmund-von Braun reaction [7,8,9], which commonly deploys excessive amounts of copper(I) cyanide and an aryl iodide at high temperature. Both methods lack today’s stringent criteria of clean synthesis that follows a greener strategy. In large-scale industrial synthesis, ammoxidation of toluene derivatives have been applied for the preparation of aryl nitriles where reaction proceeds at high temperature (300–550 °C) in the presence of a heterogeneous catalyst. Nevertheless, as the aforementioned procedures suffered from harsh reaction conditions [10,11,12], efforts are being made to address safety concerns, and to develop mild and efficient methods for the synthesis of aryl nitriles. 

The cyanation of aryl halides is a traditional strategy for C–C coupling and the preparation of aryl nitriles with transition-metal-catalyzed reactions has been an important pathway in synthetic organic chemistry. Takagi et al. [13], for the first time, disclosed Pd(II)-catalyzed cyanation of aryl halides with KCN about 40 years ago. Mechanistic studies revealed the main shortcoming of the reaction owing to excess dissociative of CN^−^ to palladium (Pd) thus initiating catalyst deactivation which poisons all the intermediates in catalytic cycle. To tackle such problems, much attention was expanded to include the addition of reducing agents or applying less soluble NaCN [14,15,16,17,18,19], trimethylsilylcyanide (TMSCN) [20], KCN [21,22,23] and Zn(CN)_2_ [24,25,26,27] salts in organic solvents; all these cyanide sources are universally toxic, due to the generation of hazardous hydrogen cyanide gas. Beller and colleagues introduced K_4_[Fe(CN)_6_] as a robust and non-toxic CN^−^ source in Pd-catalyzed coupling reactions [28] which was followed by many others using DDQ [29], *N*-cyano-*N*-phenyl-*p*-toluenesulfonamide [30], ethyl cyanoacetate [31], K_3_[Fe(CN)_6_] and K_4_[Fe(CN)_6_] [32,33,34,35,36] to name a few as safer reagents deployed in modern “cyano” chemistry.

Variety of catalytic systems constructed from palladium homogeneous catalysts have been successfully employed in the cyanation reaction due to their availability, low cost and environmental benignity [28,32,33,34,35]. Some of these methods are effective with good yields, but others suffer from one or more drawbacks such as the use of expensive ligands, prolonged reaction time, the use of homogeneous catalysts which cannot be easily recovered, and harsh reaction conditions procedures. Clearly, the development of air and thermally stable heterogeneous nanocatalysts with high activity and broad substrate tolerance is needed to allow reactions to be carried out by using K_4_[Fe(CN)_6_] as a non-toxic CN^−^ source under ligand-free conditions, then to broader industrial use of the reaction.

Metal-based nanoparticles (MNPs) have become topics of great current interest due to their potential applications in many diverse areas of science, industry, medicine and pharmaceuticals, *etc.*, especially for catalysis [37,38,39,40]. The physical techniques and chemical methods are severely applied for the synthesis of MNPs. Although, most of these methods are rather expensive and suffer from various environmental and health side effects which restrict their application in medicinal chemistry processes besides difficulty in purification and low yield [41,42,43]. Therefore, improvements leading to simplify the preparative methods for MNPs is of great importance.

Our strategy entailed the generation of palladium nanoparticles (Pd NPs) using *Cucurbita pepo* leaf extract. In view of the high propensity of metal NPs to agglomerate, solid supports have been usually applied [44,45,46,47,48] which in this study was replaced by coral reef to anchor Pd NPs. Thus, biogenically synthesized Pd/coral reef nanocomposite, assembled by a facile and eco-friendly method, has been introduced in this unprecedented cyanation reaction using a plant extract.

The *Curcurbiteae* family, a very large group with approximately 130 genera and 800 species, is widely used as food and herbal remedies around the world and they contain numerous phyto-constituents belonging to the categories of alkaloids, flavonoids, and fatty acids (palmitic-, oleic- and linoleic acids) [49,50,51,52,53,54,55]. We envisioned the leaves of the plant being an excellent source of bioreducers to biosynthesize Pd NPs nanocatalyst.

**Scheme 2:**
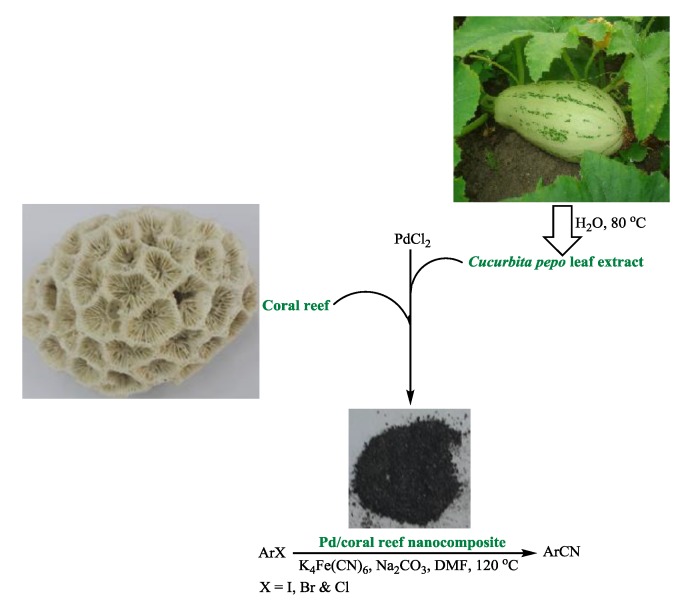
Preparation of Pd/coral reef nanocomposite using *Cucurbita pepo* leaf extract and its application for the synthesis of aryl nitriles using K_4_[Fe(CN)_6_].

In continuation of our previous efforts on the heterogeneous nanocatalysts [44,45,46], herein, for the first time, we report a convenient method for the synthesis of aryl nitriles under ligand-free conditions using K_4_[Fe(CN)_6_] as a safe source of cyanide and Pd NPs/coral reef nanocomposite as a heterogeneous catalyst (Scheme 2). This catalyst was prepared using *Cucurbita pepo* plant extract as a reducing media and fully characterized by various techniques. Moreover, an overall investigation of the effective parameters on the cyanation reaction such as solvent, bases, catalyst loading and reaction time, is described.

## 2. Experimental

### 2.1. Apparatus and Analysis

All chemicals used in the current study were obtained from Merck (Darmstadt, Germany) and Sigma-Aldrich Chemical (Sigma-Aldrich, M6250, St. Louis, MO, USA) Companies and were used without further purification. The natural (dead) coral reef used in this study originated from Persian Gulf, Iran. FT-IR (Fourier-Transform Infrared Spectroscopy) spectra were recorded using KBr pellets on a Varian model 640 spectrophotometer (Agilent Technologies, Santa Clara, CA, USA). ^1^H NMR and ^13^C NMR spectra were recorded on a Bruker Avance III 400 spectrometer (Bruker, Billerica, MA, USA) at 400 and 100 MHz, respectively. Melting points were measured on a BUCHI 510 melting point apparatus (Derwood, MD, USA) that are uncorrected. The catalyst characterization was performed by using various characterization techniques including FTIR, XRD (X-ray Diffraction), SEM (Scanning Electron Microscopy) and EDS (Energy Dispersive X-ray Spectroscopy) analysis. A Philips model X’Pert-Pro MRD diffractometer (Amsterdam, The Netherland) with a Ni-filtered Cu Kα source (λ = 0.15418 nm) was used to perform X-ray diffraction (XRD) measurements. The chemical composition of the prepared nanocomposite was performed using EDS (Energy Dispersive X-ray Spectroscopy) performed in a FESEM (Field Emission Scanning Electron Microscopy, TESCAN MIRA3-XMU, Brno-Kohoutovice, Czech Republic).

### 2.2. Preparation of the Cucurbita Pepo Leaf Extract

Fifty grams of dried powdered leaves of *Cucurbita pepo* and 300 mL double distillated water were well mixed on a magnetic heating stirrer at 80 °C for 30 min, the mixture was then centrifuged (7000 rpm) and filtered. Finally, the extract was kept at refrigerator for subsequent use.

### 2.3. Bioreduction of Pd Ions and Synthesis of Pd NPs

Ten milliliters of the extract were added dropwise to 50 mL of 0.005 M PdCl_2_ solution at 80 °C in an open glass vessel. Bioreduction process to form the nano palladium (Pd°) was completed in 5 min as color changed to dark brown; the reaction being monitored by UV-Vis spectroscopy (Shimadzu, Kyoto, Japan). Then the colored solution was centrifuged at 7000 rpm for 45 min to accomplish separation.

### 2.4. Biological Preparation of Pd/Coral Reef Nanocomposite Using Cucurbita Pepo Leaf Extract

The Pd/coral reef nanocomposite was fabricated by immobilization of Pd NPs on coral reef surface. The powdered coral reef, 1.0 g of powder was dispersed in 250 mL of the aqueous extract for 15 min at 80 °C under vigorous stirring. After that, 0.1 g of PdCl_2_ dissolved in 10 mL water and 1 mL of hydrochloric acid (37%) was added dropwise to the mixture and magnetically stirred and heated at 80 °C for 3 h. The prepared nanocomposite was centrifuged, washed with ethanol and dried in an oven and then characterized.

### 2.5. General Procedure for Synthesis of Aryl Nitriles

In a 150 mL flat bottom flask fitted with a magnetic stirrer, a mixture of aryl halide (1.0 mmol), K_4_[Fe(CN)_6_] (0.2 mmol), potassium carbonate (1.0 mmol) and nanocatalyst (0.05 g) were stirred in DMF (5.0 mL) at 120 °C and the progress of reaction was monitored using TLC. The catalyst was separated using filtration after ending the process. After completion of the reaction (as monitored by TLC), 5.0 mL of water was added then the reaction mixture was diluted using ethyl acetate (EtOAc) with vigorous stirring. The organic layer was separated, dried over MgSO_4_, filtered and the solvent removed under vacuum to afford the desired crude products. The crude product was purified by recrystallization with EtOAc and *n*-hexane to provide pure product.

## 3. Result and Discussion

Aryl nitriles were simply prepared from aryl halide in high yields by using inexpensive, readily available and non-toxic K_4_[Fe(CN)_6_], catalyzed via biosynthesis Pd/coral reef nanocomposite. The basic concept of bioreduction was followed that exploits the use of plant extract as a reducing agent and efficient stabilizer to convert metal ions to metal NPs; the synthesis of nanocomposite benefits from the combination of *Cucurbita pepo* leaf extract and coral reef as inexpensive sources. The leaf extract was characterized and then Pd NPs were separately synthesized using the *Cucurbita pepo* leaf extract as described below.

### 3.1. Characterization of Cucurbita Pepo Leaf Extract and Biosynthesized Pd Nanoparticle

**Scheme 3:**
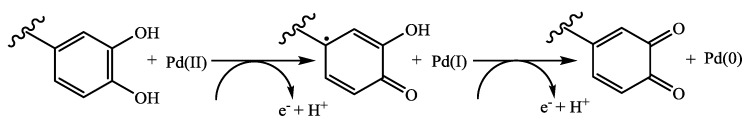
Proposed mechanism for the synthesis of Pd nanoparticles (NPs).

Although the exact mechanism for the biosynthesis of metal nanoparticles (MNPs) using plant extracts has not been confirmed, in view of the already established criteria [56,57,58,59], polar hydroxyl groups are responsible for the synthesis of MNPs [60]. Scheme 3 depicts a plausible mechanism for the bioreduction of Pd(II) ions to Pd NPs using *Cucurbita pepo* leaf extract.

**Figure 1:**
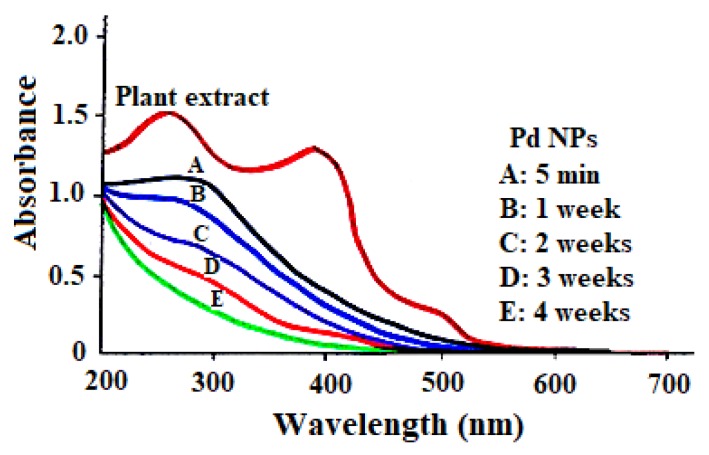
UV-Vis spectrum of plant extract and Pd NPs at different times.

The UV spectrum of the *Cucurbita pepo* leaf extract (Figure 1) shows specified signals at around 400 nm (bond Ι) and 260 nm (bond ΙΙ) due to the cinnamoyl and benzoyl systems, respectively, referring to the π → π* transitions of polyphenolics.

The UV-vis spectrum of biosynthesized Pd NPs shows the effect of surface Plasmon resonance following the appearance of maximum wavelength around 295 nm. The stability study of nanoparticles revealed that they are stable for more than 4 weeks because the wavelength of nanoparticles shows no significant deviation or disruption during this time. This stability may be due to possible adsorption of antioxidant phytochemicals on nanosurfaces, thus preventing their decomposition and deformation processes for extended periods of time.

**Figure 2:**
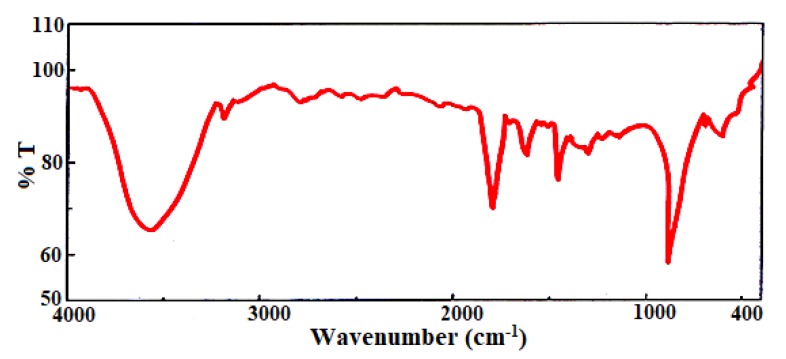
FT-IR spectrum of Pd NPs.

Figure 2 shows the FT-IR signals of Pd NPs synthesized by *Cucurbita pepo* plant extract. The main signals around 3550, 1745 and 1435–1575 cm^−1^ are assigned to the OH, carbonyl group (C=O) and C=C aromatic ring vibrations, respectively which clearly confirm the presence of plant phytochemicals on the surface of Pd NPs and their effect on protection and stability of nanoparticles.

**Figure 3:**
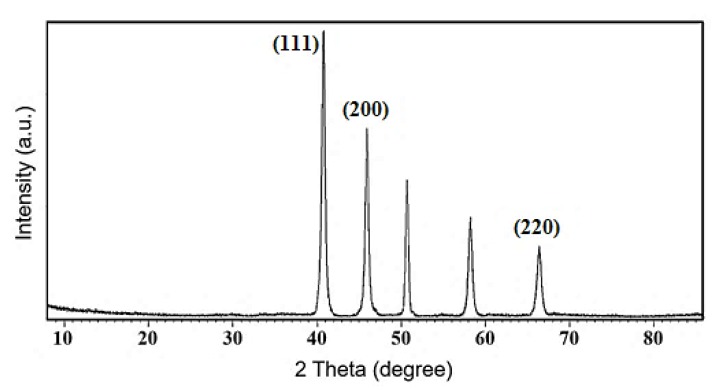
X-ray Diffraction (XRD) pattern of Pd NPs.

The XRD pattern of synthesized Pd NPs (Figure 3) exhibited a crystalline structure for the sample with peaks at 40.13° (111), 46.4° (200) and 66.5° (220), which precisely pertain to the signals of Pd NPs as corroborated by previous reports [61].

### 3.2. Characterization of Pd/Coral Reef Nanocomposite

Pd NPs, synthesized via bioreduction of Pd^2+^ ions to Pd° with assurance of stability and feasibility of their recovery, were immobilized on coral reef surface using an aqueous extract of *Cucurbita pepo* leaves. Pd/coral reef nanocomposite was confirmed by FT-IR, FE-SEM, EDS and elemental mapping.

**Figure 4:**
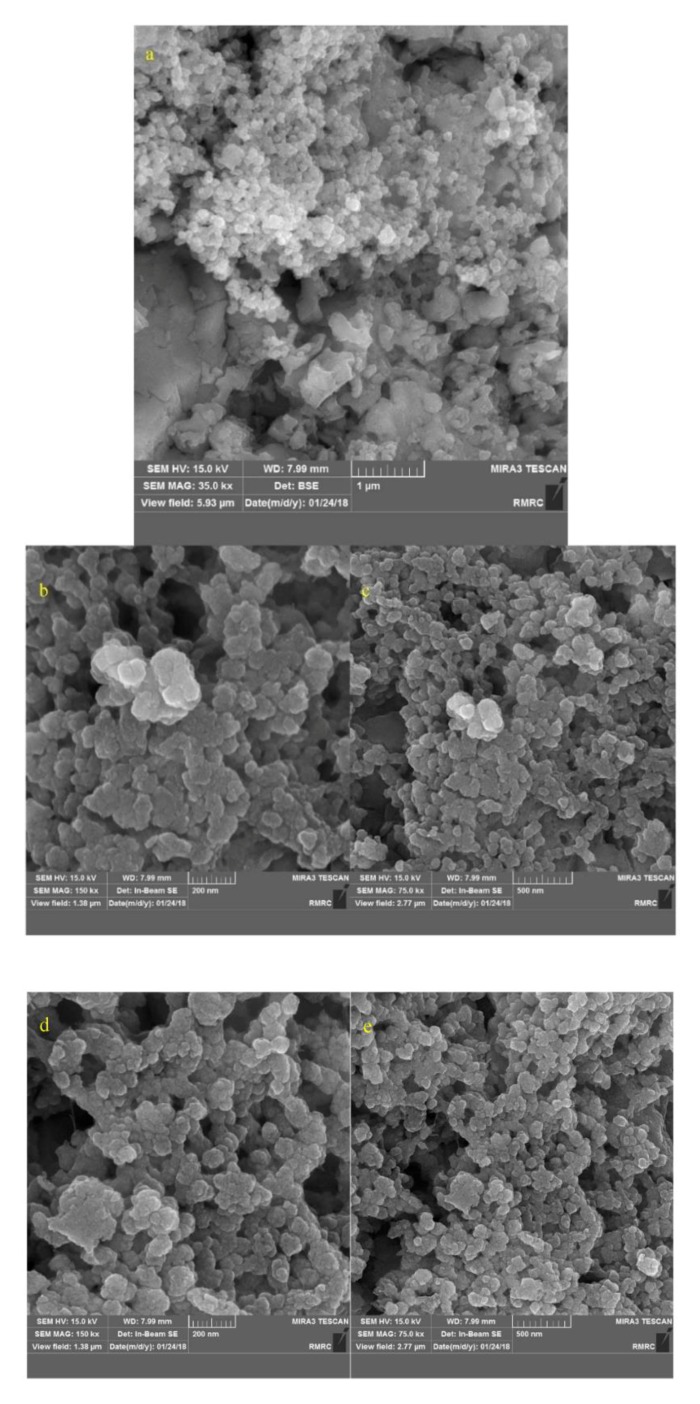
FE-SEM images of Pd/coral reef nanocomposite (**a**–**c**) and recycled Pd/coral reef nanocomposite (**d**,**e**).

**Figure 5:**
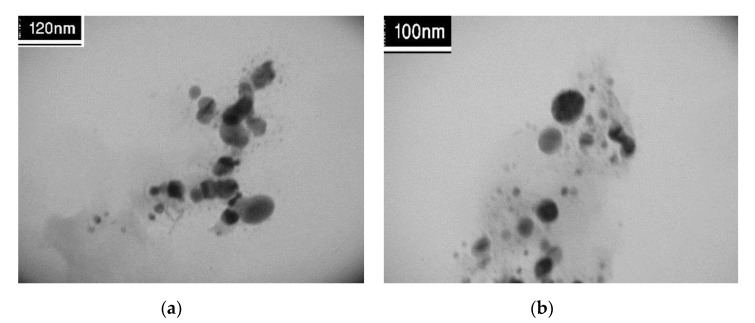
TEM images of Pd/coral reef nanocomposite. (**a**) 120 nm; (**b**) 100 nm.

**Figure 6:**
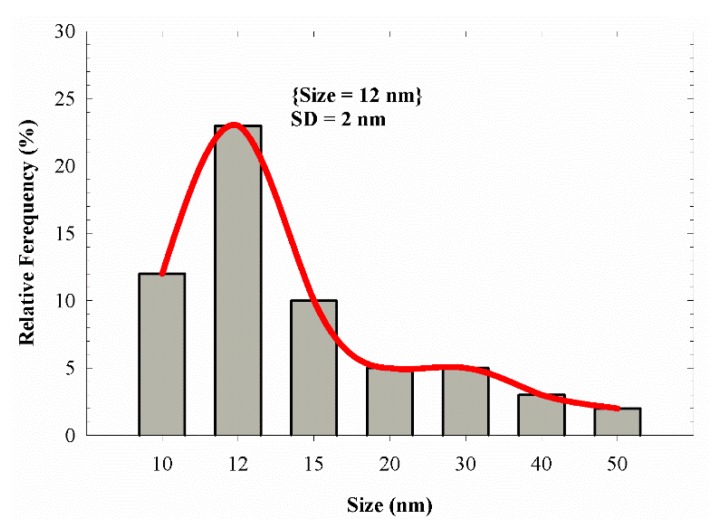
Size distribution histogram of Pd/coral reef nanocomposite.

The surface morphology and size of the as-prepared Pd/coral reef nanocomposite was examined by FE-SEM (Figure 4) and Transmission Electron Microscopy (TEM, Figure 5); the structure of the nanocatalyst was found to be well-organized pure spherical form with the monodispersity of the Pd NPs being clearly observed. Figure 6 shows the Pd NP size distributions; TEM images and histogram indicate that the average size of the Pd NPs is about 12 nm.

**Figure 7:**
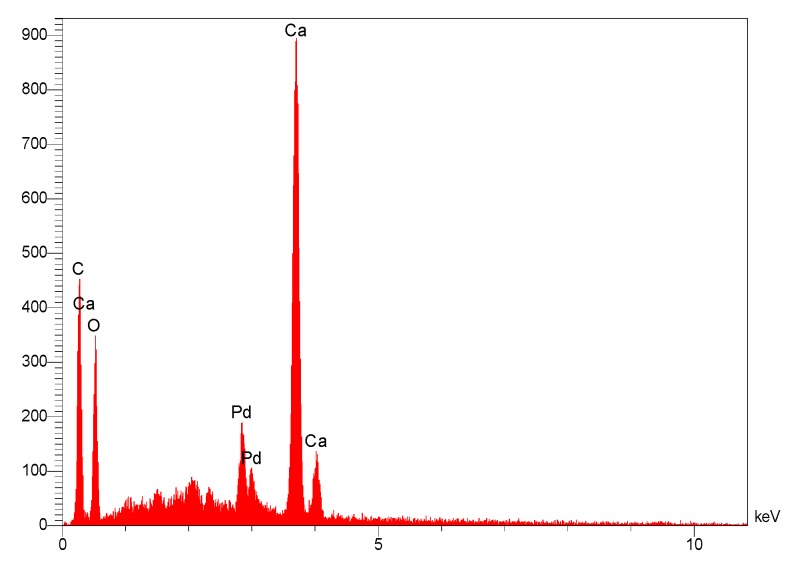
EDS spectrum of Pd/coral reef nanocomposite.

**Figure 8:**
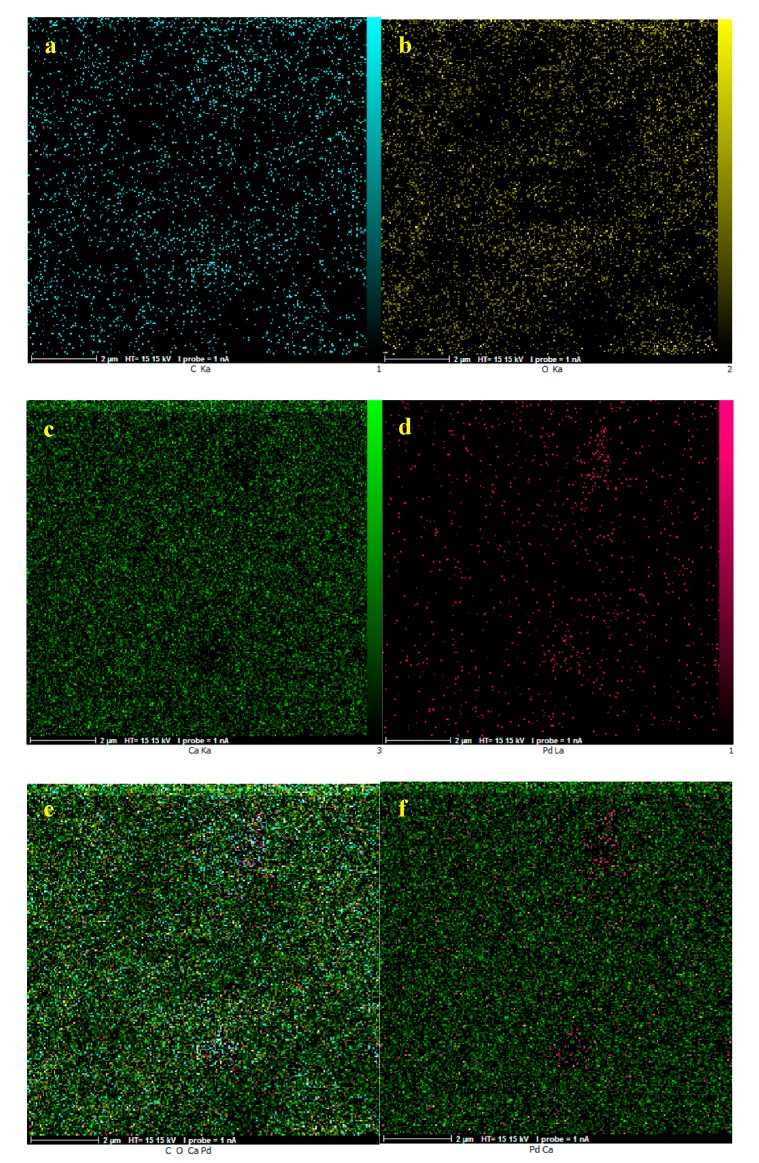
EDS elemental mapping of Pd/coral reef nanocomposite (**a**: C; **b**: O; **c**: Ca; **d**: Pd; **e**: C, O, Ca and Pd; **f**: Pd and Ca).

Further reaffirmation of the surface composition was attained by examining its composition using EDS analysis. The EDS spectrum in Figure 7 shows the presence of the C, Ca, O and Pd with the presence of Pd being endorsed by elemental mapping images (Figure 8).

**Figure 9:**
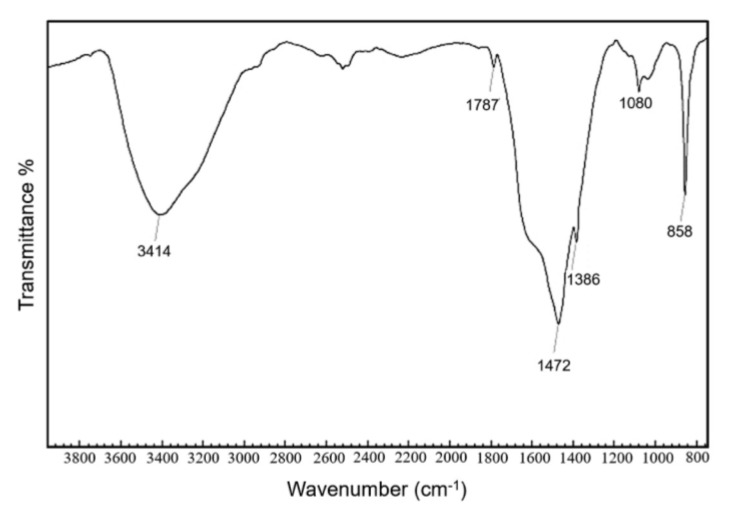
FT-IR spectrum of Pd/coral reef nanocomposite.

In order to further elucidate the presence of functional groups in the phytosynthesized nanocomposite, FT-IR analysis was recorded in the range of 400–4000 cm^−1^. As shown in Figure 9, the positions of the observed peaks are approximately similar to the corresponding peaks in the spectrum of *Cucurbita pepo* plant extract. The FT-IR spectrum of Pd/coral reef nanocomposite shows peaks at 3414, 1787, 1472, 1386, 1080, 856 cm^−1^ represent the OH functional groups, carbonyl group (C=O), stretching C=C aromatic ring, C–OH stretching vibrations and monosubstituted, *ortho-* or *meta*-disubstituted aromatic compounds vibrations, respectively. This indicates that organic compounds from the extract are adsorbed on the surface nanocatalyst through π-electron interaction; flavonoid and other phenolic compounds comprising the extract being mainly responsible for the reduction Pd^2+^ ions.

### 3.3. Catalytic Performance of the Pd/Coral Reef Nanocomposite in the Cyanation of Aryl Halides

A safe and efficient method is mandatory to generate the aryl–CN bonds for compounds used in drug development program which is adequately addressed by this work; a non-toxic cyanide source in the presence of Pd/coral reef biocatalyst was deployed. Initial experiments were conducted to determine the optimum reaction conditions (Table 1), using a model reaction between iodobenzene (1.0 mmol), K_4_[Fe(CN)_6_] (0.2 mmol), in the presence of varying amounts of the Pd/coral reef nanocomposite as catalyst and assorted solvents and bases (1.0 mmol). The reaction in the absence of Pd/coral reef nanocatalyst, produced no benzonitrile product (Table 1, entry 1). Apparently, the reaction was influenced significantly by the solvent and base deployed; the reaction was examined in solvents such as DMF (dimethylformamide), DMSO (dimethyl sulfoxide), toluene, H_2_O and *N*-methyl-2-pyrrolidone (NMP) in the presence of various bases (K_2_CO_3_, Et_3_N, NaF, Na_2_CO_3_ and KOAc). The use of DMF as solvent and K_2_CO_3_ as base gave the excellent yield (Table 1, entry 2). As shown in Table 1, catalyst was necessary for the cyanation of iodobenzene and the best results were obtained in the presence of 0.05 g of the Pd/coral reef nanocomposite (Table 1, entry 2). Decreasing the catalyst amount from 0.05 to 0.03 g decreases the product yield (Table 1, entry 11). The study further revealed that when the catalyst amount was increased from 0.05 to 0.08 g, the reaction time and yield did not show much changes (Table 1, entry 12).

The general scope of the cyanation reaction was next examined using a variety of aryl halides bearing numerous electron-donating or electron-withdrawing groups under optimized conditions and the results are summarized in Table 2. A wide variety of aryl iodides, aryl bromides and aryl chlorides were transformed into their corresponding substituted aryl nitriles in good to excellent yields. The reaction was tolerated by various functional groups such as methoxy, nitrile, nitro and hydroxyl functionalities; the nature of the substituent on the aryl halides did not affect the reaction yield including the steric effects of the *ortho*-substituents (Table 2, entry 5). As an example, among the electron-poor nitrogen heterocycles, 3-iodo- and 4-iodopyridines produced the corresponding products in good yields (Table 2, entries 9 and 10) and the reaction displayed selectivity for 1-chloro-3-iodobenzene and 1-chloro-4-iodobenzene (Table 2, entries 11 and 12). In the scenario when two I or Br groups were present, 1,4-diiodobenzene, 1,3-dibromobenzene and 1,4-dibromobenzene, (Table 2, entries 8, 15 and 16) interestingly afforded the double-addition product.

All products were obtained in good to excellent yields and characterized by FT-IR, ^1^HNMR, ^13^CNMR spectra and melting point. FT-IR spectra showed one sharp peak between 2225–2360 cm^−1^ for the CN stretching band. The formation of products was also confirmed by ^1^HNMR and ^13^CNMR spectra.

Table 3 compares the results of the present study with previously reported findings [62,63,64,65,66,67,68,69,70,71,72,73,74,75,76] for the synthesis of 4-methoxybenzonitrile. It is clearly indicated that Pd/coral reef nanocomposite is an efficient catalyst displaying excellent catalytic activity under ligand-free conditions and affording the high yield of 4-methoxybenzonitrile under short reaction time, at low catalyst loadings (Table 3). Buchwald and co-workers developed two effective protocols for cyanation of 4-methoxybromobenzene and 4-methoxychlorobenzene using K_4_Fe(CN)_6_ in the presence of palladacycle precatalyst and ligands such as *t*-BuXPhos and XPhos in excellent yields [75,76]. However, our procedure does not require any ligand for the cyanation of aryl halides. Additionally, the Pd/coral reef nanocomposite can be synthesized, via a simpler method using *Cucurbita pepo* leaf extract as a stabilizing and reducing agent without any hazardous, toxic and expensive chemicals and ligands, and the catalyst could be recovered and recycled for multiple uses. Finally, coral reef as a natural support and the plant extracts utilized the local sustainable resources originated from Persian Gulf, Iran.

### 3.4. Reusability and Stability of Catalyst

**Figure 10:**
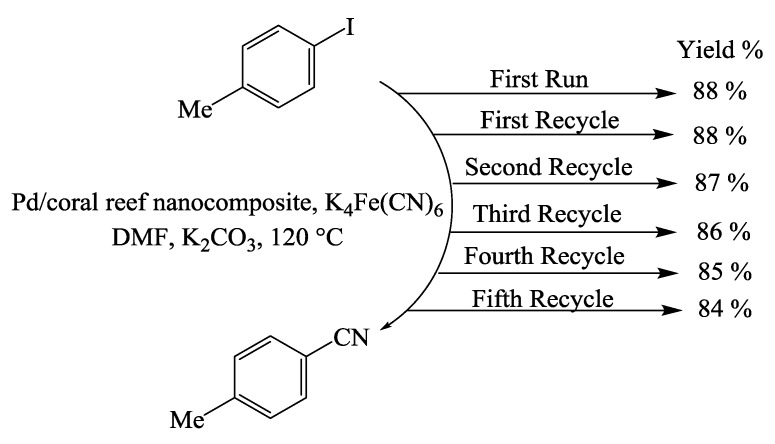
Reusability of Pd/coral reef nanocomposite for cyanation of 4-iodotoluene using K_4_Fe(CN)_6_.

**Figure 11:**
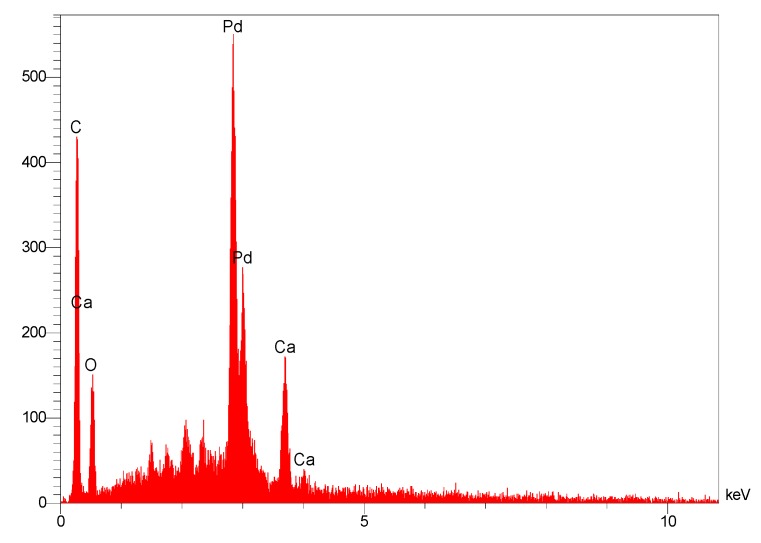
EDS spectrum of recycled Pd/coral reef nanocomposite.

The reusability of the catalysts is one of the most important aspect; it makes them useful for commercial applications, especially for precious metal use. The Pd/coral reef nanocomposite performed under heterogeneous conditions and the recovery studies were performed by conducting the cyanation reaction of 4-iodotoluene with K_4_Fe(CN)_6_ under the optimized reaction conditions. After completion of the reaction, the catalyst was separated from the reaction mixture by filtration, washed with distillated water and ethanol, dried in oven at 100 °C for 1 h and reused for the next reaction. It was interesting to observe that the catalyst can be reused up to 5 cycles with no loss of activity (Figure 10). The recovered catalyst, after five cycles, was examined by FE-SEM (Figure 4d,e) and EDS (Figure 11) analysis and the results revealed good stability of the Pd/coral reef nanocomposite in the consecutive cyanation cycles; the chemical composition and size of Pd NPs did not reveal any significant changes. To check Pd/coral reef nanocomposite heterogeneity, the leaching phenomenon was investigated by using ICP-AES (Inductively Coupled Plasma Atomic Emission Spectroscopy (Perkin Elmer 5300V, Akron, OH, USA) analysis of the resulting solution of reaction. The content of the Pd within Pd/coral reef nanocomposite, as determined by ICP-AES, was found to be 0.2 mmol/g. A hot filtration study for the cyanation reaction of 4-iodotoluene with K_4_Fe(CN)_6_ was accomplished and catalyst, immediately at the end of the reaction, was separated. The results of heterogeneity test confirm that the leaching of Pd species during reaction progress is low and Pd/coral reef nanocomposite is a heterogeneous catalyst in nature. According to the obtained results, it was revealed that less than 0.2% of the palladium was observed in the solution during the cyanation reaction.

## 4. Conclusions

In summary, we have developed a simple procedure for the cyanation of aryl halides using K_4_[Fe(CN)_6_] in the presence of Pd/coral reef nanocomposite as an efficient and recyclable heterogeneous nanocatalyst. The catalyst is obtainable via bioreduction of Pd(II) to Pd(0) NPs and its immobilization achieved on the coral reef surface as a natural support by using aqueous extract of the leaves of *Cucurbita pepo* as stabilizing and reducing agent without employing hazardous chemicals. The synthesized catalyst exhibits excellent catalytic activity for the synthesis of aryl nitriles in high yields, with a wide range of aryl halides, including electron-donating and electron-withdrawing groups. In comparison with traditional cyanation of aryl halides, our protocol provides an eco-friendlier benign and practical organic process, especially since nanocomposite were synthesis by biologically process using local resources. This protocol offers several advantages such as simple work-up procedure, high product yields, ease of preparation; separation and reusability of catalyst from the reaction mixture bodes well for its applications. Finally, in keeping with sustainability principles, the described strategy uses the readily available local resources (dead coral reef and abundant plant) for the assembly of the nanocomposite catalyst that can be exploited for synthetic transformations under greener conditions.

## Figures and Tables

**Table 1:** Pd/coral reef-catalyzed cyanation of iodobenzene using K_4_Fe(CN)_6_ at 120 °C under different conditions ^a^.

Entry	Pd/Coral Reef (g)	Solvent	Base	Time (h)	Yield (%) ^b^
1	0	DMF	K_2_CO_3_	7	0
2	0.05	DMF	K_2_CO_3_	2	88
3	0.05	DMF	NaF	7	29
4	0.05	DMF	KOAc	3	79
5	0.05	DMF	Et_3_N	7	26
6	0.05	DMF	Na_2_CO_3_	3	75
7	0.05	DMSO	K_2_CO_3_	2	85
8	0.05	NMP	K_2_CO_3_	7	28
9	0.05	Toluene	K_2_CO_3_	10	18
10	0.05	H_2_O	K_2_CO_3_	10	15
11	0.03	DMF	K_2_CO_3_	2	64
12	0.08	DMF	K_2_CO_3_	2	88

^a^ Reaction conditions: Iodobenzene (1.0 mmol), K_4_Fe(CN)_6_ (0.2 mmol), base (1.0 mmol), DMF (5.0 mL), 120 °C; ^b^ Isolated yields are after work-up.

**Table 2:** Cyanation of aryl halides with K_4_Fe(CN)_6_ in the presence of the Pd/coral reef nanocomposite ^a^.

Entry	Aryl Halide	Product	Time (h)	Yield (%) ^b^	TOF (h^−1^)
	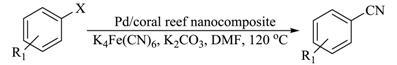				
1	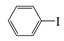	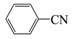	2	88	44,000
2	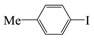	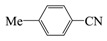	2	88	44,000
3	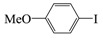	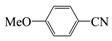	2	89	44,500
4	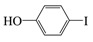	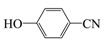	2	90	45,000
5	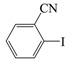	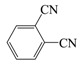	2	92	46,000
6	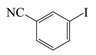	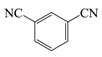	2	91	45,500
7	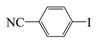	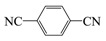	2	92	46,000
8	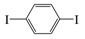	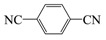	2	93^c^	46,500
9	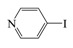	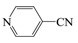	2	91	45,500
10	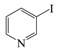	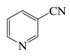	2	92	46,000
11	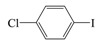	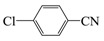	2	91	45,500
12	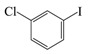	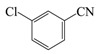	2	92	46,000
13	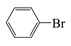	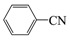	3	86	28,666
14	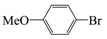	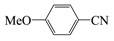	3	89	29,666
15	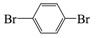	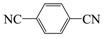	3	90^c^	30,000
16	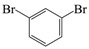	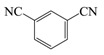	3	91^c^	30,333
17	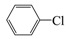	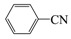	6	80	13,333
18	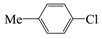	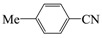	6	83	13,833
19	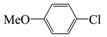	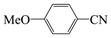	6	82	13,666

^a^ Reaction conditions: Catalyst (0.05 g containing 0.001 mol% of Pd), aryl halides (1.0 mmol), K_4_Fe(CN)_6_ (0.2 mmol), K_2_CO_3_ (1.0 mmol), DMF (5.0 mL), 120 °C; ^b^ Isolated yields are after work-up; ^c^ The reaction was carried out in the presence of 0.4 mmol of K_4_Fe(CN)_6_.

**Table 3:** Comparison of the Pd/coral reef nanocomposite with other previously reported catalysts in the synthesis of 4-methoxybenzonitrile.

Entry	Reaction Conditions	Time	Yield (%) ^a^	Ref.
1	*p*-CH_3_OC_6_H_4_I, Pd/CuO NPs, K_4_Fe(CN)_6_, K_2_CO_3_, DMF, 120 °C	15 h	88	[62]
2	*p*-CH_3_OC_6_H_4_Br, CuCN, L-proline, DMF, 120 °C	45 h	81	[63]
3	*p*-CH_3_OC_6_H_4_I, Pd(OAc)_2_, 1,1-bis(diphenylphosphino)ferrocene, HCONH_2_, POCl_3_, MW, N_2_, 160 °C	50 min	78	[64]
4	*p*-CH_3_OC_6_H_4_CH_2_OH, Cu(ClO_4_)_2_.6H_2_O, TMSN_3_, DDQ, DCE, 60 °C	4 h	82	[65]
5	*p*-CH_3_OC_6_H_4_CHO, Cu(NO_3_)_2_, NH_3_, O_2_, DMSO, 80 °C	5 h	86	[66]
6	*p*-CH_3_OC_6_H_4_I, CuI, HCONH_2_, PPh_3_, POCl_3_, N_2_, 140 °C	24 h	83	[67]
7	*p*-CH_3_OC_6_H_4_I, Cu(NO_3_)_2_.3H_2_O, HCON(Me)_2_, HOAc, TBHP, DMF, air, 140 °C	48 h	87	[68]
8	*p*-CH_3_OC_6_H_4_B(OH)_2_, CuI, PhCH_2_CN, TBHP, air, DMAc, 130 °C	20 h	72	[69]
9	*p*-CH_3_OC_6_H_4_I, [Rh(cod)Cl]_2_, Xantphos, HCONH_2_, POCl_3_, N_2_, 135-140 °C	24 h	85	[70]
10	*p*-CH_3_OC_6_H_4_I, CuF_2_, Li_2_CO_3_, CO(NH_2_)_2_, 1,10-phenanthroline, O_2_, DMSO, 150 °C	36 h	87	[71]
11	*p*-CH_3_OC_6_H_4_I, Pd-BNPs, K_4_Fe(CN)_6_, K_3_PO_4_, DMF, 120 °C	2 h	88	[72]
12	*p*-CH_3_OC_6_H_4_I, Cu(I)-HAP, K_4_Fe(CN)_6_, KF, DMF, 120 °C	15 h	80	[73]
13	*p*-CH_3_OC_6_H_4_Br, ZnO-Pd NPs, K_4_Fe(CN)_6_, KF, DMF, 130 °C	14 h	76	[74]
14	*p*-CH_3_OC_6_H_4_Cl, palladacycle precatalyst, XPhos, K_4_Fe(CN)_6_, KOAc, dioxane/H_2_O (1:1), 110 °C	1 h	95	[75]
15	*p*-CH_3_OC_6_H_4_Br, palladacycle precatalyst, *t*-BuXPhos, Zn(CN)_2_, THF/H_2_O (1:5), r.t.	18 h	97	[76]
16	*p*-CH_3_OC_6_H_4_I, Pd/coral reef, K_4_Fe(CN)_6_, K_2_CO_3_, DMF, 120 °C	2 h	89	This work
17	*p*-CH_3_OC_6_H_4_Br, Pd/coral reef, K_4_Fe(CN)_6_, K_2_CO_3_, DMF, 120 °C	3 h	89	This work

^a^ Isolated Yield.
